# Sexual Orientation– and Gender Identity–Affirming Activities Provided in Primary Care

**DOI:** 10.1001/jamanetworkopen.2025.0392

**Published:** 2025-03-10

**Authors:** Ellesse-Roselee L. Akre, Ching-Wen Wendy Yang, Greta R. Bauer, Matthew Brian Mackwood, A. James O’Malley, Elliott S. Fisher, Karen E. Schifferdecker

**Affiliations:** 1 Johns Hopkins University Bloomberg School of Public Health, Baltimore, Maryland; 2 Center for Health Systems Effectiveness, Oregon Health & Science University, Portland; 3 University of Minnesota Twin Cities, Minneapolis; 4 Dartmouth College Department of Community & Family Medicine, Lebanon, New Hampshire; 5 Geisel School of Medicine at Dartmouth, Lebanon, New Hampshire; 6 The Dartmouth Institute for Health Policy and Clinical Practice, Lebanon, New Hampshire; 7 The Dartmouth Institute, Lebanon, New Hampshire

## Abstract

**Importance:**

Addressing health disparities in lesbian, gay, bisexual, transgender, and queer (LGBTQ+) communities has been a significant policy focus for more than a decade. Ensuring access to safe, respectful primary health care from knowledgeable clinicians is crucial for reducing health inequalities.

**Objective:**

To assess the engagement of primary care practices in LGBTQ+ affirming care activities and identify practice characteristics associated with increased engagement.

**Design, Setting, and Participants:**

This cross-sectional study used data from the National Survey of Healthcare Organizations and Systems II, which focused on primary care practices in the US and was administered between June 2022 through February 2023. Participants were primary care physicians and practice managers. Percentages were weighted for national representation. Data were analyzed from November 2023 to December 2024.

**Exposure:**

Practice characteristics including practice size, ownership, federally qualified health center (FQHC) designation; percentage of patients enrolled in Medicare; percentage of patients enrolled in Medicaid; having accountable care organization (ACO) contracts for Medicare, Medicaid, or commercial insurance; rurality; census region; and LGBTQ+ Equality Score by state policy.

**Main Outcomes and Measures:**

Sexual orientation– and gender identity– (SOGI) affirming activities, a composite variable reflecting activity specific to SOGI patients.

**Results:**

Among 1245 practices in the sample (38.4% response rate), 923 (77.40%) collected data on gender identity, 921 (75.62%) on sexual orientation, and 742 (65.83%) on patients’ pronouns. Only 412 practices (34.42%) provided LGBTQ+ training for clinicians and 480 practices (39.20%) provided training for staff. Practices designated as FQHCs had 3.16 (95% CI, 4.60 to 19.73) percentage points higher probability of engaging in all SOGI-affirming activities) compared with non-FQHCs (*P* = .001). Practices with a Medicaid payer mix at least 50% had 3.28 (95% CI, 1.19 to 5.36) percentage points higher probability than practices with less than 50% Medicaid payer mix (*P* = .002), and participation in a Medicaid ACO was associated with 4.26 (95% CI, 0.78 to 7.73) percentage points increased probability compared with nonparticipants (*P* = .02). Each 1-unit increase in the state-level LGBTQ+ Equality Score was associated with 1.07 (95% CI, 0.28 to 1.85) percentage points higher probability of engaging in all SOGI-affirming activities (*P* = .02). High performance was negatively associated with practice rurality (average marginal effect, −16.00 [95% CI, −29.72 to −2.28]; *P* = .02), and rural practices were less likely to provide appropriate referrals (average marginal effect, −15.47 [95% CI, −29.22 to −1.72]; *P* = .03).

**Conclusions and Relevance:**

These findings suggest there is a need for federal and state mandates to require LGBTQ+ health care and competency training for health professionals and standardized data collection on SOGI, and these may be particularly important in rural regions. These measures are essential to evaluate practice performance and address health disparities effectively.

## Key Points

**Question:**

How engaged are primary care practices in care activities to affirm sexual orientation and gender (LGBTQ+) identity for lesbian, gay, bisexual, transgender, and queer patients, and what practice characteristics are associated with increased engagement?

**Findings:**

This cross-sectional study among 1245 primary care practices found that while 77% of practices collected data on gender identity and 76% on sexual orientation, only 34% and 39% provided LGBTQ+ training for clinicians and staff, respectively.

**Meaning:**

These findings suggest that there is a significant gap in training and data utilization by LGBTQ+ identity in primary care practices, underscoring the need for mandated training and standardized data collection to address health disparities effectively.

## Teaser

This cross-sectional study uses data from the National Survey of Healthcare Organizations and Systems II to examine primary care practices’ engagement with care to affirm the sexual orientation and gender identity of lesbian, gay, bisexual, transgender, and queer patients and practice characteristics associated with increased engagement.

## Introduction

For more than a decade, there has been increasing interest in addressing the health care needs of lesbian, gay, bisexual, transgender, and/or queer (LGBTQ+) people. Reports from the Institute of Medicine, Healthy People 2020; National Academies of Sciences, Engineering and Medicine; and the Agency for Healthcare Research and Quality have highlighted the need to better understand and address LGBTQ+ health care needs.^1,2,3^ These populations have an elevated risk of experiencing certain health conditions and correspondingly a reduced life expectancy of up to 12 years when they are exposed to social stigma, structural discrimination, and the subsequent inequities in access to social determinants of health that they create.^4^ People who identify as LGBTQ+ experience higher rates of anxiety and depression and higher rates of disability and functional limitation as older adults,^5,6^ creating a need for more health care while having limited access.^7,8,9^

LGBTQ+-affirming care is an approach to health care that validates and supports the identities, experiences, and needs of LGBTQ+ individuals. Affirming care activities seek to create a safe and inclusive environment for LGBTQ+ patients, which can have positive associations with their health outcomes, mental -well-being, and patient-clinician communication.^10^ LGBTQ+ affirming care activities, which include collecting data on sexual orientation and gender identity (SOGI), creating a welcoming environment, LGBTQ+-friendly or protective policies and trainings, use of appropriate language, providing appropriate services, and providing suitable referrals, are essential for creating safe and inclusive environments that can benefit all patients.^11^ For instance, environments that ensure safety and privacy for patients who choose to disclose their SOGI identity and the use of a patients’ correct pronouns provide appropriate and accessible care for all patients regardless of SOGI and have been demonstrated to improve the health care experience for LGBTQ+ patients.^12,13,14^ Even as a growing number of patients, irrespective of race, ethnicity, or age, are willing to disclose their SOGI to health care practitioners, studies suggest clinicians are apprehensive about collecting SOGI data, citing inadequate training, lack of understanding of its relevance to care delivery, and their belief that patients would not be comfortable sharing.^15,16^

While not to be conflated with gender-affirming medical care, such as hormone therapy or gender-affirming surgical procedures, social gender affirmation—including the authentic use of names and pronouns—is a critical component of LGBTQ+-affirming care activities.^17,18^ This aligns with the broader conceptualization of affirming care, as outlined by Reisner et al,^19^ emphasizing respect and recognition of individuals’ identities in all aspects of care.^19^

In the wake of the disparate impact of COVID-19 on LGBTQ+ communities^13,20,21,22,23,24,25,26,27^ and politicization of their health services,^28^ there are increasing calls for systemwide efforts focused on achieving health equity for LGBTQ+ people. High-quality primary care has been shown to form the foundation of a high-performing health system^29^ and consistently results in better population health outcomes and reduced inequities.^30^ This suggests that access to primary care with systems and training in care activities for LGBTQ+ patients is a critical step in improving their overall health and outcomes.

To our knowledge, no national data exist on primary care practices’ efforts to ensure high-quality care for LGBTQ+ patients. This survey assessed US primary care practices’ engagement in LGBTQ+-affirming activities and identified practice-level factors associated with these efforts, aiming to inform policies that promote health equity and affirming care.

## Methods

This cross-sectional study was approved as exempt with a waiver of signed consent by Dartmouth College institutional review board because any disclosure of the human participants’ responses outside the research would not reasonably place the participants at risk of criminal or civil liability or be damaging to the participants’ financial standing, employability, educational advancement, or reputation. This study follows the Strengthening the Reporting of Observational Studies in Epidemiology (STROBE) reporting guideline for cross-sectional studies.

### Data

Data for the project come from wave II of the National Survey of Healthcare Organizations and Systems (NSHOS II), a nationally representative survey of primary care practices.^31^ Data were collected from June 2022 through February 2023. The survey included 52 items that captured various aspects of primary care practices’ size, ownership, payment models, practice capabilities, and other practice characteristics. We used a stratified sampling design to draw samples of primary care practices with at least 3 primary care physicians, including longitudinal respondents to NSHOS I,^32^ federally qualified health centers (FQHCs), and practices that were not FQHCs. Non-FQHCs were further stratified by ownership and area deprivation level. Practices were identified using IQVIA’s commercial OneKey database. FQHC status was determined using the Health Resources and Services Administration’s Health Center Delivery Service dataset and practice self-report, number of physicians was retrieved from OneKey,^33^ and area deprivation was determined through the University of Wisconsin’s Neighborhood Atlas.^34^

### Measures

#### Outcome Measure Development

Items to assess attention to and processes related to SOGI-affirming activities were initially developed by E.-R. L. A., a health services researcher with expertise in health equity and LGBTQ+ health care access and utilization, and K. E. S., a medical anthropologist and survey expert, with subsequent input from all research team members. We constructed items based on care recommendations that affirm individuals who identify as LGBTQ+ and the Human Rights Campaign’s Health Equity Index (HEI), a national benchmarking tool that evaluates health care facilities’ policies and practices related to the equity and inclusion of their LGBTQ+ patients, visitors, and employees.^10,35,36,37,38^ The resulting 8 items capture a practice’s ability to engage in LGBTQ+-affirming service activities across 4 constructs: SOGI data collection, appropriate service referrals, use of SOGI data collected, and training. We piloted the items with 13 primary care clinicians and practice managers to ensure comprehensibility and relevance.

#### Outcome

Our main outcome, SOGI-affirming activities, is an ordinal count of 8 possible practice activities. Survey items were equally weighted, with 1 point per activity. We scored missing items as a 0, since we believe nonresponse is a surrogate for an activity not being performed. SOGI-affirming activity total scores ranged from 0 to 8. The internal consistency of the survey items using Cronbach α was 0.76, representing a moderate to high level of internal consistency for the SOGI-affirming activity items (eAppendix 1 in Supplement 1). To simplify reporting, we created a binary outcome measure based on SOGI-affirming activity scores, defining high performers—consistent with Human Rights Campaign’s HEI—as practices engaging in 80% (≥6) of the activities.^38^

We analyzed associations between construct items to assess associations between engagement in SOGI-affirming activities and practice characteristics. The constructs include SOGI data collection (no data collected, 1, 2, or all data points), providing appropriate referrals, created by dichotomizing an ordinal variable (not at all, a little or some, or quite a lot), use of SOGI data (no use, use by system or practice level, or use by system and practice level), and SOGI training (no training, training for clinicians or staff, or trainings for clinicians and staff).

#### Exposures

We used several practice characteristics in the analysis: practice size (very small, <4 physicians; small, 5-9 physicians; medium, 10-20 physicians; and large, ≥21 physicians), practice ownership (independent or other ownership), FQHC (FQHC/look-alike or no designation), percentage of patients enrolled in Medicare (≥50% or <50%), percentage of patients enrolled in Medicaid (≥50% or <50%), having an accountable care organization (ACO) contract for Medicare (yes or no), Medicaid (yes or no), and/or commercial insurers (yes or no). FQHC status, Medicaid and Medicare payer mix, and ACO participation may influence the provision of SOGI-affirming activities by reflecting a practice’s capacity to serve underserved populations, focus on inclusivity in care delivery, and alignment with value-based care models that emphasize comprehensive, equitable services. In addition to practice characteristics, we included rurality (small town or smaller vs nonrural) based on Rural-Urban Commuting Areas,^39^ census region (West, Midwest, South, and Northeast), and LGBTQ+ Equality Score by state policy (negative, low, fair, medium, or high overall score), a measure of state level LGBTQ+ policies, to provide insight into the sociopolitical context of where care is being delivered.^40^

### Statistical Analysis

All analyses are weighted to account for our unequal probability sampling design and subsequent nonresponse.^31^ Details on the weighting for the sample can be found in eAppendix 2 in Supplement 1. We conducted a descriptive analysis comparing high performers (≥6 activities) to others using χ^2^ tests and calculated rates for each activity and the SOGI-affirming activity composite measure. Univariate ordinal and binary logistic regression models assessed associations between the SOGI-affirming activity composite, constructs, and practice characteristics, with average marginal effects (AMEs) using robust SEs for calculating *P* values and CIs. We used Stata software version 18 (StataCorp) for the analysis. *P* values were 2-sided, and statistical significance was set at *P* < .05. Data were analyzed from November 2023 to December 2024.

## Results

We contacted 3498 practices and received 1540 responses, for a 38.4% response rate. After excluding 137 duplicate surveys and 151 surveys with significant missing data, the final analysis included 1245 practices. Sampling and nonresponse weights were used to account for differential group representation in the sampling strategy and adjust measures to more accurately represent the findings for the national sample frame.

The Figure provides an overview of performance on the individual SOGI items. We found that 923 practices (weighted, 77.40%) collected data on gender identity, 921 practices (weighted, 75.62%) collected data on sexual orientation, and 742 practices (weighted, 65.83%) collected data on patients’ pronouns. Only 412 practices (weighted, 34.42%) and 480 practices (weighted, 39.20%) offered LGBTQ+ competency trainings for clinicians and staff, respectively. A total of 250 practices (weighted, 19.89%) reviewed performance measures by patient sexual orientation, and 274 practices (weighted, 28.99%) reviewed measures at the system level. Approximately half of practices (691 [weighted, 55.77%]) provided referrals to physicians specialized in treating LGBTQ+ patients.

**Figure.**
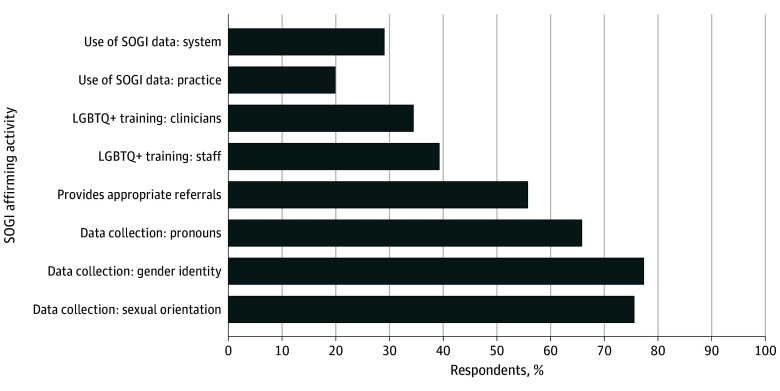
Weighted Percentage of Practices Participating in Sexual Orientation– and Gender Identity– (SOGI) Affirming Services Data were collected from the 2022 National Survey of Healthcare Organizations and Systems II. LGBTQ+ indicates lesbian, gay, bisexual, transgender, and queer.

Table 1 presents the characteristics of participating practices by SOGI scores and percentile ranking. Analyses are shown with unweighted numbers to show the true size of our sample alongside weighted proportions to reflect the inferred characteristics of the national sample. We identified 330 practices (weighted, 28.68%) as high performers. We found differences between the high-performing practices and other practices by FQHC status, census region, rurality, state-level LGBTQ+ Equality Score, percentage of patients enrolled in Medicaid, and Medicaid ACO participation. The proportion of high performers was highest in the Midwest (86 practices [30.43%]) and lowest in the South (75 practices [18.40%]). Nearly half of the high performing practices were in states with high LGBTQ+ Equality Scores (156 practices [45.35%]). High-performing practices participated in a mean of 6.68 (SE, 0.08; 95% CI, 6.52-6.84) activities, compared with 2.82 (SE, 0.10; 95% CI, 2.62-3.02) activities for all other practices.

**Table 1.** Primary Care Practices by Characteristics and SOGI-Affirming Activity Scores

Characteristic	Practices, No. (weighted %)		
	High performers (n = 330)	Others (n = 915)	Total (N = 1245)
Practice size (No. of physicians)			
Very small (<4)	119 (45.07)	349 (28.05)	468 (40.06)
Small (5-9)	106 (34.72)	320 (36.57)	426 (36.04)
Medium (10-20)	49 (11.79)	144 (15.74)	193 (14.61)
Large (≥21)	52 (8.42)	91 (9.64)	143 (9.29)
Practice ownership			
Independent physician	151 (35.59)	487 (44.33)	638 (41.87)
Other (system or hospital)	153 (64.41)	392 (55.67)	545 (58.13)
FQHC status^a^			
Not FQHC	232(77.67)	792 (88.25)	1024 (85.22)
FQHC or FQHC look-alike	98 (22.33)	123 (11.75)	221 (14.78)
Rurality^b^			
Not rural	312 (95.75)	817 (91.12)	1129 (92.45)
Rural	18 (4.25)	98 (8.88)	116 (7.55)
Census region^b^			
Northeast	68 (29.08)	171 (18.57)	239 (21.59)
Midwest	86 (30.43)	246 (32)	332 (31.55)
South	75 (18.4)	293 (32.66)	368 (28.57)
West	101 (22.08)	205 (16.77)	306 (18.29)
Overall state-level LGBTQ+ Equality Score^b^			
Negative	19 (3.34)	93 (10.87)	112 (8.71)
Low	58 (16.37)	209 (27.12)	267 (24.03)
Fair	49 (22.51)	166 (19.12)	215 (20.09)
Medium	44 (12.43)	98 (12.21)	142 (12.27)
High	156 (45.35)	338 (30.68)	494 (34.89)
Medicare payer population, %			
<50	247 (97.17)	867 (98.32)	991 (98.02)
≥50	13 (2.83)	12 (1.68)	25 (1.98)
Medicaid payer population, %^c^			
<50	208 (83.92)	689 (91.85)	897 (89.74)
≥50	61 (16.08)	80 (8.15)	141 (10.26)
Participation in Medicare ACO contract			
Not now/never	78 (23.13)	252 (27.01)	330 (25.93)
Yes	216 (76.87)	593 (72.99)	809 (74.07)
Participation in Medicaid ACO contract^a^			
Not now/never	120 (37.57)	507 (59.3)	627 (53.25)
Yes	174 (62.43)	331 (40.7)	505 (46.75)
Participation in Commercial ACO contract			
Not now/never	117 (35.07)	379 (40.89)	496 (39.27)
Yes	177 (64.93)	457 (59.11)	634 (60.73)

Abbreviations: ACO, accountable care organization; FQHC, federally qualified health center; LGBTQ+, lesbian, gay, bisexual, transgender, and queer.

^a^ *P* < .001.

^b^ *P* < .05.

^c^ *P* < .01.

Table 2 presents associations of SOGI-affirming activity overall and construct scores with individual practice characteristic using either a univariate ordinal logistic regressions or bivariate logistic regressions reporting the AME at each level of the independent variable. FQHCs had 15.46 (95% CI, 8.32 to 22.61) percentage points higher probability of being a high performing practice than non-FQHCs (*P* < .001). Practices with a Medicaid payer mix at least 50% had 14.81 (95% CI, 6.21 to 23.40) percentage points higher probability than those with a payer mix of less than 50% (*P* < .001), and those participating in a Medicaid ACO had 17.07 (95% CI, 7.45 to 26.70) percentage points greater probability of being high performers (*P* < .001). A 1-unit increase in the LGBTQ+ Equality Score was associated with 5.88 (95% CI, 2.84 to 8.91) percentage points increased probability of being a high performing practice (*P* < .001). High performance was negatively associated with practice rurality (AME, −16.00 [95% CI, −29.72 to −2.28]; *P* = .02).

**Table 2.** SOGI-Affirming Activities Engagement by LGBTQ+ Affirming Care Constructs and Overall Performance by Practice Characteristics

Activity	AME (95% CI)^a^										
	Practice ownership (n = 1183)	FQHC status (n = 1245)	Rurality (n = 1245)	% Payer population		Participation in			Practice size (n = 1230)	Census region (n = 1245)	State-level LGBTQ+ Equality Score (n = 1230)
				Medicare (n = 1016)	Medicaid (n = 1038)	Medicare ACO (n = 1139)	Medicaid ACO (n = 1132)	Commercial ACO (n = 1130)			
High performer (≥6 vs ≤5 activities)	0.0734 (−0.0242 to 0.1710)	0.1546 (0.0832 to 0.2261)^b^	−0.16 (−0.2972 to −0.0228)^c^	0.1028 (−0.0734 to 0.2790)	0.1481 (0.0621 to 0.2340)^b^	0.0413 (−0.0678 to 0.1504)	0.1707 (0.0745 to 0.2670)^b^	0.0495 (−0.0522 to 0.1513)	−0.0307 (−0.0784 to 0.0169)	−0.0278 (−0.0767 to 0.0212)	0.0588 (0.0284 to 0.0891)^b^
SOGI data points collected, No.											
None	−0.0578 (−0.1169 to 0.0013)	−0.1012 (−0.1683 to −0.0342)^d^	0.0392 (−0.0376 to 0.1161)	−0.0105 (−0.1308 to 0.1098)	−0.1336 (−0.2153 to −0.0520)^d^	−0.07 (−0.1348 to −0.0051)^c^	−0.1265 (−0.1965 to −0.0565)^b^	−0.057 (−0.1226 to 0.0086)	0.0099 (−0.0251 to 0.0449)	0.0044 (−0.0243 to 0.0332)	−0.0304 (−0.0528 to −0.0080)^d^
1	−0.0138 (−0.0306 to 0.0031)	−0.0238 (−0.0397 to −0.0079)^d^	0.0093 (−0.0098 to 0.0283)	−0.0027 (−0.0329 to 0.0276)	−0.0335 (−0.0550 to −0.0120)^d^	−0.0165 (−0.0340 to 0.0009)	−0.0287 (−0.0465 to −0.0110)^d^	−0.0131 (−0.0290 to 0.0029)	0.0023 (−0.0060 to 0.0107)	0.001 (−0.0059 to 0.0080)	−0.0071 (−0.0131 to −0.0011)^c^
2	−0.0168 (−0.0376 to 0.0041)	−0.0303 (−0.0476 to −0.0131)^b^	0.0121 (−0.0129 to 0.0370)	−0.0031 (−0.0389 to 0.0326)	−0.0402 (−0.0619 to −0.0184)^b^	−0.0208 (−0.0426 to 0.0009)	−0.0356 (−0.0534 to −0.0178)^b^	−0.0171 (−0.0377 to 0.0036)	0.0031 (−0.0078 to 0.0140)	0.0014 (−0.0076 to 0.0103)	−0.0094 (−0.0167 to −0.0021)^c^
All	0.0883 (−0.0067 to 0.1834)	0.1553 (0.0617 to 0.2490)^d^	−0.0606 (−0.1807 to 0.0595)	0.0163 (−0.1700 to 0.2026)	0.2073 (0.0946 to 0.3200)^b^	0.1073 (0.0064 to 0.2083)^c^	0.1908 (0.0958 to 0.2858)^b^	0.0872 (−0.0128 to 0.1872)	−0.0153 (−0.0695 to 0.0388)	−0.0068 (−0.0514 to 0.0377)	0.0469 (0.0128 to 0.0810)^d^
Provide appropriate referrals (vs not able to provide referrals)^a^	−0.0408 (−0.1455 to 0.0638)	0.1199 (0.0237 to 0.2160)^c^	−0.1547 (−0.2922 to −0.0172)^c^	0.0306 (−0.2157 to 0.2768)	0.1546 (0.0348 to 0.2744)^c^	−0.0275 (−0.1426 to 0.0875)	0.0564 (−0.0570 to 0.1697)	−0.0436 (−0.1522 to 0.0649)	−0.0341 (−0.0872 to 0.0191)	−0.0061 (−0.0555 to 0.0433)	0.0421 (0.0060 to 0.0782)^c^
Use of SOGI data											
None	−0.0833 (−0.1874 to 0.0208)	−0.0282 (−0.1222 to 0.0658)	0.0695 (−0.0851 to 0.2241)	−0.0122 (−0.2069 to 0.1826)	−0.0408 (−0.1497 to 0.0681)	−0.0945 (−0.2161 to 0.0272)	−0.2208 (−0.3147 to −0.1268)^b^	−0.1225 (−0.2298 to −0.0152)^c^	0.0739 (0.0248 to 0.1230)^d^	0.0433 (−0.0063 to 0.0929)	−0.0128 (−0.0489 to 0.0234)
By system or practice	0.0595 (−0.0138 to 0.1328)	0.0202 (−0.0478 to 0.0882)	−0.0498 (−0.1595 to 0.0599)	0.0088 (−0.1317 to 0.1492)	0.0292 (−0.0499 to 0.1084)	0.0661 (−0.0191 to 0.1513)	0.153 (0.0956 to 0.2104)^b^	0.0853 (0.0114 to 0.1593)^c^	−0.0527 (−0.0871 to −0.0183)^d^	−0.031 (−0.0652 to 0.0033)	0.0091 (−0.0166 to 0.0349)
By system and practice	0.0238 (−0.0088 to 0.0565)	0.008 (−0.0182 to 0.0342)	−0.0197 (−0.0653 to 0.0259)	0.0034 (−0.0510 to 0.0578)	0.0116 (−0.0187 to 0.0418)	0.0284 (−0.0102 to 0.0670)	0.0677 (0.0201 to 0.1154)^d^	0.0372 (−0.0002 to 0.0745)	−0.0212 (−0.0387 to −0.0038)^c^	−0.0123 (−0.0285 to 0.0038)	0.0036 (−0.0069 to 0.0141)
SOGI training											
None	−0.0571 (−0.1576 to 0.0434)	−0.1872 (−0.2687 to −0.1058)^b^	0.191 (0.0532 to 0.3287)^d^	−0.066 (−0.2620 to 0.1301)	−0.1915 (−0.2881 to −0.0949)^b^	0.0155 (−0.0924 to 0.1234)	−0.164 (−0.2646 to −0.0633)^d^	−0.0011 (−0.1055 to 0.1033)	0.0164 (−0.0363 to 0.0691)	0.0197 (−0.0289 to 0.0683)	−0.0752 (−0.1054 to −0.0450)^b^
Clinicians or staff	0.0058 (−0.0038 to 0.0154)	0.0179 (0.0042 to 0.0315)^c^	−0.018 (−0.0320 to −0.0040)^c^	0.0078 (−0.0162 to 0.0317)	0.0219 (0.0045 to 0.0394)^c^	−0.0017 (−0.0136 to 0.0103)	0.0166 (0.0048 to 0.0284)^d^	0.0001 (−0.0108 to 0.0111)	−0.0016 (−0.0066 to 0.0035)	−0.0019 (−0.0064 to 0.0026)	0.0068 (0.0030 to 0.0107)^b^
Clinicians and staff	0.0513 (−0.0401 to 0.1427)	0.1694 (0.0983 to 0.2404)^b^	−0.1729 (−0.3001 to −0.0458)^d^	0.0582 (−0.1143 to 0.2307)	0.1695 (0.0863 to 0.2528)^b^	−0.0138 (−0.1098 to 0.0821)	0.1473 (0.0548 to 0.2399)^d^	0.001 (−0.0925 to 0.0944)	−0.0148 (−0.0625 to 0.0329)	−0.0178 (−0.0620 to 0.0264)	0.0684 (0.0400 to 0.0967)^b^

Abbreviations: ACO, accountable care organization; AME, average marginal effect; FQHC, federally qualified health center; LGBTQ+, lesbian, gay, bisexual, transgender, and queer; SOGI, sexual orientation and gender identity.

^a^ Binary logistic regression.

^b^ *P* < .001.

^c^ *P* < .05.

^d^ *P* < .01.

Practices with FQHC designation had higher probabilities of collecting all data points, such as pronouns (AME, 15.53 [95% CI, 6.17-24.90]; *P* < .001), providing appropriate referrals (AME, 11.99 [95% CI, 2.37-21.60]; *P* = .02), and offering training for both clinicians and staff (AME, 16.94 [95% CI, 9.83-24.04]; *P* < .001). Practices where at least 50% of patients were covered by Medicaid were more likely to collect all data points (AME, 20.73 [95% CI 9.46-32.00]; *P* < .001), provide referrals (AME, 15.46 [95% CI, 3.48-27.44]; *P* < .001), and train clinicians and staff (AME, 16.95 [95% CI, 8.63-25.28]; *P* < .001). Similarly, participation in Medicaid ACOs was associated with increased likelihood of collecting all data points (AME, 19.08 [95% CI, 9.58-28.58]; *P* < .001), using SOGI data at both the practice and system levels (AME, 6.77 [95% CI, 2.01-11.54]; *P* = .005), and training clinicians and staff (AME, 19.08 [95% CI, 5.48-23.99]; *P* = .002). Participation in Medicare ACOs was also positively associated with collecting all data points (AME, 10.73 [95% CI, 0.64-20.83]; *P* = .04). Higher state-level scores were associated with collecting all data points (AME, 4.69 [95% CI, 1.28-8.10]; *P* = .007), providing appropriate referrals (AME, 4.21 [95% CI, 0.60-7.82]; *P* = .02), and training clinicians and staff (AME, 6.84 [95% CI, 4.00-9.67]; *P* < .001).

Practices in rural areas were less likely to provide appropriate referrals (AME, −15.47 [95% CI, −29.22 to −1.72]; *P* = .03) or train clinicians and staff (AME. −17.29 [95% CI, −30.01 to −4.58]; *P* = .008). Additionally, the number of physicians in the practice was negatively associated with using SOGI data at the practice and system levels (AME, −2.12 [95% CI, −3.87 to −0.38]; *P* = .02).

Lastly, we calculated the change in the probability of a practice engaging in each level of SOGI-affirming activity by practice characteristic (Table 3). We found that FQHCs had 3.16 (95% CI, 4.60-19.73) percentage points higher probability of engaging in all SOGI-affirming activity activities compared with non-FQHCs (*P* = .001). Practices with Medicaid payer mix of 50% or more had 3.28 (95% CI, 1.19-5.36) percentage points higher probability of participating in all SOGI-affirming activities (*P* = .002), and practices that participated in a Medicaid ACO had 4.26 (95% CI, 0.78-7.73) percentage points higher probability. Lastly, each 1-unit increase in the state-level LGBTQ+ Equality Score was associated with an increase in probability of engaging in all SOGI-affirming activities by 1.07 (95% CI, 0.28-1.85) percentage points (*P* = .008).

**Table 3.** Practice Characteristics and Sexual Orientation and Gender Identity Affirming Activities at Each Level

Overall activities, No.	AME (95% CI)^a^										
	Practice ownership (n = 1183)	FQHC status (n = 1245)	Rurality (n = 1245)	% Payer population		Participation in			Practice size (n = 1230)	Census region (n = 1245)	State-level LGBTQ+ Equality Score (n = 1230)
				Medicare (n = 1016)	Medicaid (n = 1038)	Medicare ACO (n = 1139)	Medicaid ACO (n = 1132)	Commercial ACO (n = 1130)			
0	−0.0248 (−0.0541 to 0.0044)	−0.0623 (−0.0993 to −0.0253)^b^	0.0433 (0.0095 to 0.0772)^c^	−0.0177 (−0.0988 to 0.0635)	−0.0719 (−0.1150 to −0.0287)^d^	−0.0238 (−0.0566 to 0.0089)	−0.0769 (−0.1184 to −0.0354)^b^	−0.0223 (−0.0547 to 0.0102)	0.0122 (−0.0039 to 0.0284)	0.0068 (−0.0089 to 0.0225)	−0.021 (−0.0334 to −0.0086)^b^
1	−0.0188 (−0.0425 to 0.0048)	−0.0466 (−0.0731 to −0.0202)^b^	0.0325 (0.0058 to 0.0591)^c^	−0.0138 (−0.0766 to 0.0490)	−0.0562 (−0.0886 to −0.0238)^b^	−0.0184 (−0.0443 to 0.0074)	−0.0577 (−0.0857 to −0.0297)^b^	−0.0172 (−0.0425 to 0.0081)	0.0088 (−0.0020 to 0.0196)	0.0051 (−0.0066 to 0.0167)	−0.0149 (−0.0230 to −0.0069)^b^
2	−0.0124 (−0.0287 to 0.0038)	−0.0315 (−0.0482 to −0.0149)^b^	0.0222 (0.0031 to 0.0413)^c^	−0.008 (−0.0441 to 0.0282)	−0.0327 (−0.0498 to −0.0155)^b^	−0.012 (−0.0301 to 0.0061)	−0.0367 (−0.0541 to −0.0193)^b^	−0.0112 (−0.0282 to 0.0058)	0.0063 (−0.0015 to 0.0140)	0.0035 (−0.0047 to 0.0116)	−0.0105 (−0.0164 to −0.0046)^b^
3	−0.0147 (−0.0353 to 0.0059)	−0.0374 (−0.0558 to −0.0191)^b^	0.0269 (0.0028 to 0.0511)^c^	−0.0099 (−0.0546 to 0.0348)	−0.0397 (−0.0600 to −0.0193)^b^	−0.0138 (−0.0348 to 0.0071)	−0.0392 (−0.0584 to −0.0201)^b^	−0.0126 (−0.0318 to 0.0066)	0.0077 (−0.0021 to 0.0174)	0.0042 (−0.0060 to 0.0144)	−0.0128 (−0.0201 to −0.0055)^b^
4	0.0016 (−0.0028 to 0.0061)	0.0035 (−0.0097 to 0.0168)	−0.0019 (−0.0102 to 0.0064)	0.0024 (−0.0097 to 0.0146)	0.0094 (−0.0087 to 0.0275)	0.0014 (−0.0034 to 0.0063)	0.0028 (−0.0109 to 0.0166)	0.0011 (−0.0034 to 0.0056)	−0.0005 (−0.0029 to 0.0019)	−0.0003 (−0.0016 to 0.0009)	0.0008 (−0.0031 to 0.0047)
5	0.0107 (−0.0023 to 0.0238)	0.0257 (0.0079 to 0.0435)^d^	−0.0182 (−0.0329 to −0.0035)^c^	0.0084 (−0.0303 to 0.0472)	0.0332 (0.0103 to 0.0562)^d^	0.0111 (−0.0047 to 0.0269)	0.0323 (0.0160 to 0.0486)^b^	0.0101 (−0.0044 to 0.0247)	−0.0051 (−0.0114 to 0.0012)	−0.0028 (−0.0092 to 0.0035)	0.0086 (0.0034 to 0.0137)^d^
6	0.022 (−0.0070 to 0.0509)	0.0557 (0.0255 to 0.0858)^b^	−0.0394 (−0.0724 to −0.0065)^c^	0.015 (−0.0536 to 0.0836)	0.06 (0.0269 to 0.0931)^b^	0.0214 (−0.0093 to 0.0522)	0.0641 (0.0365 to 0.0917)^b^	0.0201 (−0.0092 to 0.0493)	−0.0111 (−0.0250 to 0.0028)	−0.0062 (−0.0203 to 0.0080)	0.0187 (0.0078 to 0.0296)^b^
7	0.0239 (−0.0075 to 0.0553)	0.0614 (0.0309 to 0.0920)^b^	−0.0433 (−0.0805 to −0.0061)^c^	0.0154 (−0.0546 to 0.0853)	0.065 (0.0332 to 0.0967)^b^	0.0211 (−0.0104 to 0.0527)	0.0687 (0.0342 to 0.1032)^b^	0.0198 (−0.0109 to 0.0504)	−0.0121 (−0.0277 to 0.0035)	−0.0068 (−0.0232 to 0.0097)	0.0205 (0.0089 to 0.0322)^b^
8	0.0125 (−0.0055 to 0.0305)	0.0316 (0.0129 to 0.0503)^b^	−0.0221 (−0.0451 to 0.0009)	0.008 (−0.0282 to 0.0442)	0.0328 (0.0119 to 0.0536)^d^	0.013 (−0.0066 to 0.0326)	0.0426 (0.0078 to 0.0773)^c^	0.0122 (−0.0075 to 0.0319)	−0.0062 (−0.0150 to 0.0027)	−0.0034 (−0.0121 to 0.0053)	0.0107 (0.0028 to 0.0185)^d^

Abbreviations: AME, average marginal effect; ACO, accountable care organization; FQHC, federally qualified health center; LGBTQ+, lesbian, gay, bisexual, transgender, and queer.

^a^ Binary logistic regression.

^b^ *P* < .001.

^c^ *P* < .05.

^d^ *P* < .01.

## Discussion

Our cross-sectional study addresses a key gap in the literature by assessing US primary care practices’ engagement in LGBTQ+-affirming activities and identifying factors associated with high engagement. We found generally high rates of SOGI data collection but limited use of these data to improve performance or train staff. Practices with FQHC or FQHC look-alike status, high Medicaid payer mixes, Medicaid ACO participation, and favorable state LGBTQ+ policy climates were significantly more likely to engage in all 8 SOGI-affirming activity items. However, only approximately half of practices provided relevant referrals for LGBTQ+ patients. Structural and policy-level factors, such as FQHC designation and Medicaid engagement, were critical factors associated with participation in SOGI-affirming activities, while rurality and larger practice size posed significant barriers. These findings highlight trends in SOGI-affirming activity engagement and emphasize the importance of supportive policies and systems in advancing LGBTQ+-affirming care.

The study found high rates of SOGI data collection in primary care, which, while not addressing discrimination or bias, is a crucial first step in understanding LGBTQ+ patient needs and improving care quality.^10,37,41^ The finding that training was scarce among practices is perhaps not surprising, given there is no national policy requiring this training, underscored by findings from a 2011 study of LGBTQ+ education in US medical schools that found these schools had 0 hours of LGBTQ+ health content during clinical years.^42^ If the Liaison Committee for Medical Education and the Accreditation Council for Continuing Medical Education included LGBTQ+ health training as a requirement for accreditation, it could reduce misinformation contributing to stigmatization and discrimination of LGBTQ+ patients in health care and increase the confidence and proficiency of clinicians so that LGBTQ+ affirming care could be more broadly considered a standard of all care rather than just specialty care. Such policies could increase access to quality LGBTQ+ care for millions of US patients.

Our finding that FQHCs were more likely to be high performers on the SOGI-affirming activities composite measure may reflect the overall mission of FQHCs to provide health care services for underserved populations or regions with quality assurance protocols.^43^ In 2016, the US Health Resources and Services Administration mandated the reporting of SOGI data by all FQHCs, which may contribute the higher scores for SOGI-affirming activities in FQHCs,^44^ albeit FQHC status was positively associated with all 4 LGBTQ+-affirming constructs. Recent studies on SOGI data collection in FQHCs have reported that nearly 70% and 74% of patient records included sexual orientation and gender identity, respectively.^44^ Additionally there are specific targeted improvements for LGBTQ+-focused care at FQHCs.^45^ Additionally FQHCs may be more aware and equipped to provide affirming care, given their focus on oppressed populations and considering that 63% of their patients are people of color.^46^ Further research is warranted to delve into the nuanced dynamics influencing SOGI inclusivity across different regions and funding types as well as to understand the effects these services have on health outcomes.

The finding that the South and rural areas had the lowest probability of having higher performing practices is troubling when considering that many LGBTQ+ people in rural areas may experience limited access to necessary health services and experience health disparities.^7,47,48^ Similarly, the associations of Medicaid payer mix and ACO contract participation with participation in SOGI-affirming activities require further investigation. It is possible that practices with a high percentage of Medicaid patients and participation in Medicaid ACO contracts may be better at engaging in SOGI-affirming activities because these practices are often more focused on addressing health disparities and improving access to care for underserved populations. Medicaid, which covers a higher proportion of low-income and marginalized individuals, including LGBTQ+ communities, may incentivize practices to implement inclusive care models that meet the diverse needs of these populations. Participation in Medicaid ACOs often emphasizes comprehensive, coordinated care and quality improvement,^49^ which can include adopting practices that affirm patients’ SOGI. These practices may be more likely to engage in SOGI-affirming activities as part of broader efforts to enhance care quality, reduce health disparities, and meet the cultural and clinical needs of their diverse patient base.

Primary care practices in states with high overall LGBTQ+ Equality Scores are likely to be better at engaging in SOGI-affirming activities due to the supportive legal and policy environment in these states. High LGBTQ+ Equality Scores often reflect state-level policies that promote LGBTQ+ rights, antidiscrimination protections, and inclusivity in health care settings. Research has shown that LGBTQ+ individuals in states with limited protections are more likely to report poor self-rated health, depression, and physical health burdens compared with their cisgender and heterosexual counterparts.^50,51^

### Limitations

As with any study, this study has some limitations. First, the LGBTQ+-affirming care questions do not capture every aspect of affirming care. In efforts to reduce survey burden, we were limited in the number of questions that we could ask of the research participants. Future surveys should develop additional items to explore other aspects of LGBTQ+-affirming care that were not included in the survey, such as the physical environment and antidiscrimination policies,^36,41^ and should assess content and quality of training for medical care, including gender-affirming primary care. Second, the study relies on self-report from each practice and is subject to recall bias and desirability bias.^52^ As health equity and LGBTQ+ health are highly politicized topics, some respondents may have overstated some of their practices to appear as though their practices are more inclusive than they are, which means that our results likely overestimate the true extent of LGBTQ+-affirming services in primary care nationally. However, given the distribution of responses, respondents did acknowledge gaps in their practice’s processes or approaches related to these topics. Additionally, while the survey response rate was modest, it is similar to other large-scales surveys that were administered during the COVID-19 pandemic, and the respondents in the sample are consistent with the sampling frame.^53,54^

## Conclusions

This cross-sectional study of LGBTQ+-affirming services in US primary care found that most practices primarily focused on collecting SOGI data and patients’ pronouns, with only slightly more than one-quarter of practices classified as high performers. While many practices collected SOGI data and provided referrals for LGBTQ+-specific services, fewer used the data or conducted staff and clinician training. Our findings highlight significant gaps in LGBTQ+-affirming activities across primary care. The study supports the effectiveness of the FQHC model in addressing LGBTQ+ needs compared with other practice types and underscores the need for national policies mandating LGBTQ+-inclusive training in medical and nursing education to reduce barriers and improve access to safe, appropriate care for millions of US patients.

## Notes

Supplement 1. **eAppendix 1.** Survey Items for Sexual Orientation and Gender Identity Affirming Services Composite Measures **eAppendix 2.** Details on Survey Weighting and Sampling Frame

Supplement 2.
Data Sharing Statement
