# Error in Funding/Support

**DOI:** 10.1001/jamanetworkopen.2025.11171

**Published:** 2025-04-15

In the Original Investigation titled “Sexual Orientation– and Gender Identity–Affirming Activities Provided in Primary Care,”^1^ published March 10, 2025, there was an error in the Funding/Support. This article has been corrected.^1^
