# Supplementary material for: Sexual Orientation– and Gender Identity–Affirming Activities Provided in Primary Care
Source: JAMA Netw Open. 2025 Mar 10;8(3):e250392. doi: 10.1001/jamanetworkopen.2025.0392 (PMC11894487; doi:10.1001/jamanetworkopen.2025.0392)
Supplement: Supplement 1. — eAppendix 1. Survey Items for Sexual Orientation and Gender Identity Affirming Services Composite Measures eAppendix 2. Details on Survey Weighting and Sampling Frame [file jamanetwopen-e250392-s001.pdf]

Supplemental Online Content

Akre ERL, Yang CWW, Bauer GR, et al. Sexual orientation– and gender identity–affirming activities provided in primary care. *JAMA Netw Open*. 2025;8(3):e250392. doi:10.1001/jamanetworkopen.2025.0392

eAppendix 1. Survey Items for Sexual Orientation and Gender Identity Affirming Services Composite Measures
eAppendix 2. Details on Survey Weighting and Sampling Frame

This supplemental material has been provided by the authors to give readers additional information about their work.

© 2025 Akre EL et al. *JAMA Network Open*.

Appendix A. Survey items for sexual orientation and gender identity (SOGI) affirming services (SAA) composite measures.

SOGI Affirming Activities.

SOGI data collection

Does your practice routinely collect information about patients':

1. Sexual orientation
   i. Yes
   ii. No
2. Gender identity (e.g., transgender, non-binary)
   i. Yes
   ii. No
3. Preferred Pronouns
   i. Yes
   ii. No

Referrals

4. To what extent is your practice prepared to refer lesbian, gay, bisexual, transgender, queer (LGBTQ) patients to clinicians who specialize in LGBTQ needs and concerns?
   a. Not at all/ a little
   b. Some/ quite a lot

© 2025 Akre EL et al. *JAMA Network Open*.

Training

Does your practice require training for clinicians and staff on the following:

5. Clinicians: Providing high quality care to LGBTQ populations
   i. Yes
   ii. No
6. Staff: Providing high quality care to LGBTQ populations
   i. Yes
   ii. No

Performance

Does your practice review its performance on delivery of preventive services stratified by the following patient characteristics?

7. Practice level
   i. Yes
   ii. No
8. System level
   i. Yes
   ii. No

© 2025 Akre EL et al. *JAMA Network Open*.

Appendix B. Details on survey weighting and sampling frame.

All analyses were conducted using survey weights to adjust for the likelihood that a practice was selected from the sampling frame of eligible practices (determined based on the 2022 population of medical practices in the US) and whether the practice participated in our survey (to account for non-response). Specifically, we surveyed 100% of Federally Qualified Health Centers (FQHCs) and a nearly equal number (166 or 167) of non-FQHCs (totaling 1000) for the six combinations of system type and the binary variable indicating whether the Federal Information Processing Standards (FIPS) decile was 8 or higher. Therefore, there were seven types of practices in total (six non-FQHCs and FQHCs).

The sampling probabilities were calculated as simple proportions, defined as the numerator divided by the number of practices in the original sampling frame (before knowing eligibility information learned through the survey) for the seven types of practices. After sampling, some practices were determined to be ineligible, resulting in seven proportions of eligible practices. Among the eligible sampled practices, a fraction responded, yielding seven proportions of respondents. Thus, the joint probability of a practice being sampled, found eligible, and responding was estimated as the product of these three proportions for each of the seven types of practices. The

© 2025 Akre EL et al. *JAMA Network Open*.

inverses of these joint probabilities were used as marginal weights in our chi-square test analyses.

© 2025 Akre EL et al. *JAMA Network Open*.
