# Supplementary material for: Sexual Orientation– and Gender Identity–Affirming Activities Provided in Primary Care
Source: JAMA Netw Open. 2025 Mar 10;8(3):e250392. doi: 10.1001/jamanetworkopen.2025.0392 (PMC11894487; doi:10.1001/jamanetworkopen.2025.0392)
Supplement: Supplement 2. — Data Sharing Statement [file jamanetwopen-e250392-s002.pdf]

Data Sharing Statement

Akre. Sexual Orientation– and Gender Identity–Affirming Activities Provided in Primary Care. *JAMA Netw Open*. Published March 10, 2025. doi:10.1001/jamanetworkopen.2025.0392

Data
Data available: Yes
Data types: Deidentified participant data
How to access data: A deidentified version of the data that supports the findings of this study are available from the author KA upon reasonable request.
When available: With publication

Supporting Documents
Document types: None

Additional Information
Who can access the data: Researchers whose proposed use of data has been approved.
Types of analyses: Secondary data analysis
Mechanisms of data availability: With a signed data access agreement.
Any additional restrictions: None
